# The Impact of Hormonal Replacement Treatment in Postmenopausal Women with Uterine Fibroids: A State-of-the-Art Review of the Literature

**DOI:** 10.3390/medicina55090549

**Published:** 2019-08-30

**Authors:** Elisa Moro, Eugenia Degli Esposti, Giulia Borghese, Federica Manzara, Margherita Zanello, Diego Raimondo, Giulia Gava, Alessandro Arena, Paolo Casadio, Maria Cristina Meriggiola, Renato Seracchioli

**Affiliations:** Gynecology and Human Reproduction Physiopathology, Dipartimento di Scienze Mediche e Chirurgiche (DIMEC), S. Orsola Hospital, University of Bologna, Via Massarenti, 13–40138 Bologna, Italy

**Keywords:** menopause, hormonal replacement therapy, uterine fibroids, abnormal uterine bleeding, postmenopausal women

## Abstract

*Background and Objectives*: Hormonal replacement therapy (HRT) is effective in treating many debilitating symptoms of menopause. However, its use in women with uterine fibroids is widely debated, based on the susceptibility of these tumors to sexual steroids. This review aims to ascertain the effects of HRT on leiomyomas development and growth in postmenopausal women. *Materials and Methods*: Electronic databases (i.e., MEDLINE, Scopus, ClinicalTrials.gov, EMBASE, Sciencedirect, the Cochrane Library at the CENTRAL Register of Controlled Trials, Scielo) were searched from January 1990 until May 2019. All English-written studies evaluating the impact of various HRT regimens on uterine leiomyomas were selected. *Results*: Seventeen papers, considering a total of 1122 participants, were included. Fifteen of these were prospective trials, of which nine were randomized controlled trials. The remaining two works were a retrospective observational trial and a retrospective case series respectively. Five studies evaluated the effects of tibolone, also comparing it with various estrogen/progestin combinations, while two were about raloxifene. Thirteen studies compared different combinations of estrogens/progestins, the most common being transdermal estrogens (used in nine studies) and medroxyprogesterone acetate at different doses (used in 10 studies). *Conclusions*: For women with uterine fibroids, the choice of the most appropriate HRT regimen is crucial to avoid leiomyomas growth and the symptoms possibly related to it. Available data are conflicting, but suggest that uterine fibroids might be influenced by HRT, without representing an absolute contraindication to hormonal replacement therapy. Women with uterine fibroids subjected to HRT should be periodically examined and hormonal treatment should be discontinued if leiomyomas appear to increase in size. Moreover, the minimal effective dose of progestin should be employed.

## 1. Introduction

Leiomyomas are the most common benign uterine neoplasms, affecting up to 80% of reproductive age women and causing morbidity in up to 30% of them [1,2,3,4]. Their exact etiology remains unclear, but some studies suggest that fibroids are monoclonal tumors originating from a single myocyte [5]. Many risk factors have been linked to leiomyomas, such as African-American ethnicity, older age, younger age at menarche and nulliparity [6,7,8]. Symptoms associated with uterine fibroids can vary widely among women, based on their size and location. Abnormal uterine bleeding, pelvic pain or pressure, bloating, urinary frequency and constipation/tenesmus are some of the most frequent complaints [9,10,11,12].

It is well established that uterine leiomyomas are hormone-dependent masses. In fact, they show a tendency to enlargement during reproductive age, have never been observed before menarche and tend to shrink after menopause [13,14]. Moreover, the administration of estrogen-progestin was shown to lead to myomas growth during reproductive age [15,16].

Fibroids growth appears to be influenced both by ovarian steroidogenesis and by estrogen produced in situ, by conversion of local androgens to estrogens by aromatase [1]. Interestingly, aromatase mRNA was detected in more than 90% of uterine fibroids, but not in the myometrium of women without them, suggesting a key-role in leiomyomas growth [1,17]. Several studies have confirmed the presence of estrogen receptors in fibromatous tissue, with a pattern that differs from normal myometrium, since mRNA expression of estrogen receptors is reportedly greater in leiomyomas [18,19]. Also, the reactivity of uterine leiomyomas to estrogens appear to be strongly related with the increase of their receptors on uterine myoma tissue [16,18]. Uterine fibroids demonstrate a growth pattern that seems to parallel the physiological changes in serum estradiol levels [14].

Progestins may also play a crucial role in the development and growth of uterine leiomyomas [20], as proved by their mitotic index, which is increased in the luteal phase of the menstrual cycle [19]. Furthermore, progesterone receptors are overexpressed in fibromatous tissue compared to healthy surrounding myometrium [21,22,23], especially in smaller fibroids [24]. The importance of progestins in leiomyomas growth is further proven by the regression of uterine fibroids under treatment with antiprogesterone drugs, such as mifepristone and ulipristal acetate.

Based on this, the use of hormonal replacement therapy (HRT) in women affected by fibroids has been widely debated, and the previous diagnosis of leiomyomas has even been considered as a relative contraindication to the prescription of HRT. In fact, uterine fibroids tend to shrink during menopause and often become asymptomatic, thus requiring no treatment in the majority of patients [13,14]. Specific therapy is reserved only to large fibroids or leiomyomas that continue to grow after menopause and, in this age group is usually represented by total hysterectomy [12].

With the increase in life expectancy, it is estimated that 1.2 billion women worldwide will reach menopause by 2030 [25]. Menopause is a physiological event in a woman’s life, but it carries a significant burden, causing unpleasant and debilitating symptoms in up to 60% of women, the most common being vasomotor symptoms, genitourinary syndrome of menopause, osteoporosis, insomnia, depression and weight gain [21,26]. Current literature suggest that menopausal symptoms might be the result of the reduction of estrogen levels and (HRT) has proven itself effective in treating many of these symptoms.

In light of this, HRT use in menopausal women with fibroids is controversial, because of the possible role of estrogens and progestins on myomas growth. Therefore, the aim of this state-of-the-art review is to revise current literature on the possible implications of HRT on leiomyomas development, growth and symptoms in postmenopausal women.

## 2. Materials and Methods

### Search Strategy and Selection Criteria

To identify relevant papers for inclusion in our literature review, a thorough search of Electronic databases (i.e., MEDLINE, Scopus, ClinicalTrials.gov, EMBASE, Sciencedirect, the Cochrane Library at the CENTRAL Register of Controlled Trials, Scielo) was performed from January 1990 until May 2019. Search terms used were the following text words: “menopause,” “uterine leiomyomas”, “hormonal replacement therapy”, “menopause hormonal therapy”, “uterine fibroids.” Only English-written papers were considered for this review. No restrictions for geographic location were applied. In addition, the reference lists of all identified articles were examined to identify studies not captured by electronic searches. The electronic search and the eligibility of the studies were independently assessed by three authors (E.D.E., G.B., F.M.). Final inclusion was decided after detailed examination of the studies. Differences were discussed, and consensus reached. We included all randomized clinical trials, retrospective studies, literature reviews and case reports and series dealing with the use of hormonal replacement therapy in menopause in patients with evidence of uterine leiomyomas.

## 3. Results

**Figure 1:**
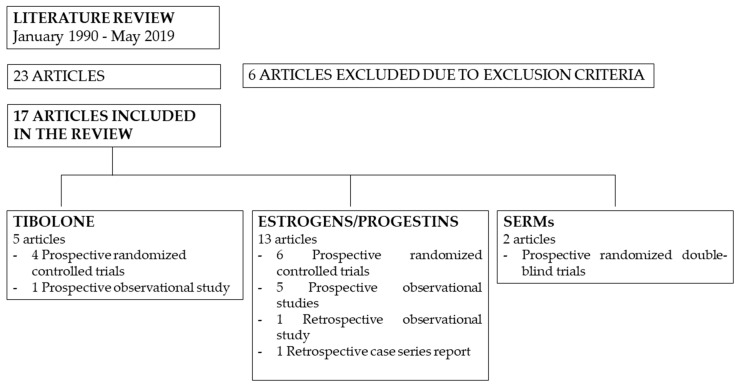
Flow chart of the literature review.

**Figure 2:**
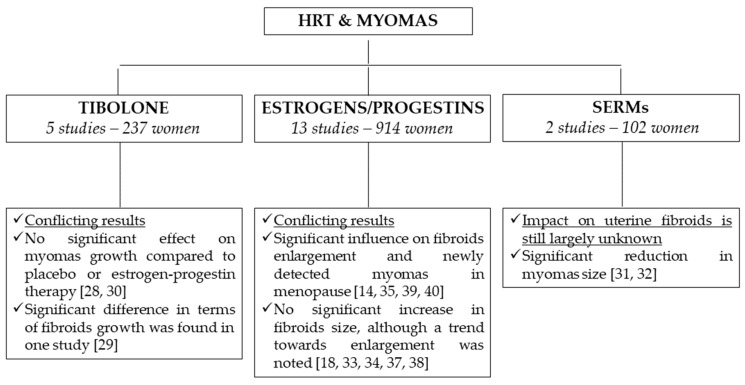
Impact of HRT on the size of uterine fibroids.

Twenty-three articles were identified as relevant papers employing the research strategy mentioned above Figure 1. Of these, seventeen articles were selected to be included in our review based on the aforementioned criteria, comprehending a total of 1122 women. In particular, 15 studies were prospective trials, of which nine were randomized controlled trials. The remaining two papers were an observational retrospective study and a retrospective case series report. Five papers analyzed the effects of tibolone, either alone [27] or compared to no treatment [19] or to various combinations of estrogens/progestogens [28,29,30], and two studies compared the effects of raloxifene to placebo [31,32]. As for estrogens, transdermal 17-β-estradiol was the most used method [14,18,29,30,33,34,35,36,37], followed by conjugated equine estrogen (CEE) [14,18,28,37,38], estradiol-17-valerate [18,35,39] and micronized estradiol [37,40]. Regarding progestogens, medroxyprogesterone acetate (MPA) was the most common, and was used at different doses: 10 mg [29,35,36,37], 5 mg [14,18,28,38,40] and 2.5 mg [14,34,37,40]. Other HRT regimens included nomegestrol acetate [33], prasteronenantate via intramuscular injection [39], micronized progesterone [18,37], cyproterone acetate [35] and norethisterone acetate [30]. Eleven studies had a follow-up period of 12 months, 2 of 36 months and one of 24 months. The remaining three studies considered periods of 6 months, 3 months and a mean period of 19.7 ± 6.3 months, respectively. Data from all articles are presented in Table 1 and summarized in Figure 2.

### 3.1. Tibolone

Tibolone is a synthetic steroid which, after conversion into active metabolites, exerts weak estrogenic, androgenic and progestogenic effects [19]. In particular, tibolone helps to preserve bone mineral density, improve female sexual function and relieve vasomotor symptoms [21]. Lacking a stimulatory effect on the endometrium, it reduces the incidence of spotting and abnormal uterine bleeding [41], and makes the concurrent administration of progestogens unnecessary.

Gregoriou et al. analyzed the influence of a 2.5 mg/day tibolone regimen in post-menopausal women with leiomyomas in two studies [19,27]. In a prospective, randomized controlled trial [19] 40 naturally menopausal women were randomized into two groups of 20 women each: group A received therapy, whereas group B did not receive any treatment. After one-year follow-up, the mean volume of fibroids in both groups was slightly reduced, but with no statistical significance. Moreover, the number of women with increased, unchanged and decreased myoma volume were comparable between the two groups. In a subsequent study [27], Gregoriou et al. analyzed the effects of the same treatment protocol on three different groups of women (based on the presence and mean diameter of uterine fibroids) over a period of 3 years. In addition to myoma mean volume, the authors evaluated also uterine blood flow patterns, measuring the pulsatility index (PI) of the uterine arteries. As found by other authors, normal uteri showed higher vascular resistances compared to fibromatous uteri, which had significantly lower PIs. After 3 years, myoma volume was not significantly different from baseline. On the other hand, after 6 months of tibolone administration, the PI value in women without fibroids showed a significant reduction when compared to baseline values, and it remained unchanged until the end of the study. In the same period, the mean PI values in women with smaller and larger fibroids increased significantly (1.62 ± 0.25 to 1.86 ± 0.20 and 1.72 ± 0.23 to 1.94 ± 0.20, *p* < 0.01). No differences were noted between the mean PI values of growing and stable fibromas in groups B and C.

In a randomized controlled trial, De Aloysio et al. [28] compared the effects of 2.5 mg/day of Tibolone to a regimen of Conjugated Equine Estrogen (CEE) 0.625 mg/day plus medroxyprogesterone acetate (MPA) 5 mg/day for 12 cycles on myomas size and patients’ bleeding patterns. According to their findings, there were no statistical differences in terms of fibroids size at the end of treatment. Moreover, women in both groups had a reduced incidence of spotting and irregular bleeding after 12 cycles compared to baseline, but this decline was more evident in the tibolone group than the CEE + MPA one (22.6% and 2.4% of the cycles vs. 29.7% and 4.7% respectively).

Fedele and colleagues [29] conducted a randomized controlled trial involving 38 menopausal women with one or more uterine myomas, all symptomatic before menopause. Women were randomly allocated to two different groups: 20 women received a transdermal estradiol-releasing system (50 mcg/day) plus oral MPA 10 mg/day, 18 women were administered tibolone 2.5 mg/day. Patients were evaluated every 3 months throughout a period of 12 months. Their data demonstrated a significant increase in myoma size and number, compared to baseline, in women treated with transdermal estradiol and MPA in the first 6 months of treatment, with a stabilization over the next 6 months. Conversely, these findings were not observed in the tibolone group.

Another study conducted by Simsek et al. [30] compared the effects of tibolone 2.5 mg/day and of sequential transdermal estradiol (50 mcg/day) and norethisterone acetate 0.25 mg/day (administered for 4 and 2 weeks/month respectively). In the tibolone arm, the mean volume of myomas increased from 18.6 to 20.1 cm^3^, whereas in the transdermal estradiol arm it increased from 23.1 to 27.3 cm^3^, without any statistical significance, neither within nor between groups. Also, there was no statistical difference in terms of frequency of myomas growth, although it was slightly higher in the transdermal treatment arm (21.4% vs. 12.5%). Moreover, three women in the tibolone group and two in the transdermal one reported abnormal uterine bleeding; endometrial biopsies did not show any relevant alterations in four women, whereas an endometrial polyp was diagnosed in a patient receiving tibolone.

### 3.2. Estrogens/Progestins

Several studies compared different regimens of HRT in postmenopausal women with uterine fibroids. Colacurci et al. [33] studied the effects of continuous transdermal 17-beta-estradiol (50 mcg/day) plus nomegestrol acetate (5 mg/day) sequentially added on leiomyomas size and uterine arteries PI over a period of 12 months. Sixty menopausal patients were divided into 3 groups based on the presence and the dimensions of uterine leiomyomas. Women with fibroids were further divided based on the eventual growth of myomas during treatment (women in groups B1 and C1 had quiescent myomas, whereas women in groups B2 and C2 had myomas growing > 20%). After a year, myoma mean volume was slightly increased both in women with smaller and larger fibroids, but with no statistical significance when compared to baseline values (24.1 ± 20.0 vs. 28.8 ± 30.0). In accordance with data available in literature, uterine arteries PI at baseline was markedly higher in healthy uteri (2.4 ± 0.5 vs. 1.7 ± 0.2 and 1.6 ± 0.3 respectively). Moreover, the baseline PI in subgroups B1 and C1 was higher than PI in subgroups B2 and C2, but was lower than that of group A, pointing out that a very low value of uterine arteries PI might be a predictor of the risk of leiomyoma growth during HRT. Interestingly, all relevant changes in terms of PI occurred during the first 3 months of treatment and were stable in the following 9 months.

In a prospective observational trial, Frigo et al. [39] studied 50 perimenopausal women who received intramuscular injections of 4 mg estradiol-valerate and 200 mg prasteronenantate every 6–10 weeks over 12 months. The evaluation of myoma size and number at baseline and at the end of treatment showed a significant increase in both variables. In particular, myomas number increased from a median of 2.2 to 3.5, and their mean diameter grew from 29.4 mm to 35.0 mm (*p* < 0.001).

In a prospective non-randomized trial, Palomba et al. [34] evaluated the effects of transdermal estradiol patches (50 mcg/day) plus oral MPA (2.5 mg/day) continuously added on women with and without uterine fibroids over 1 year. Particularly, 92 menopausal women were divided into three groups: group A consisted of women with no more than 2 intramural or subserosal fibroids < 20 mm, group B consisted of women with no more than 2 intramural-subserosal fibroids > 20 mm, and group C included controls without uterine fibroids. Women in groups A and C were submitted to HRT, whereas women in group B received calcium carbonate tablets as placebo. Fibroids in group A and B showed a non-significant growth overtime (141.7 ± 37.8 vs. 147.5 ± 53.3 and 150.3 ± 58.7 vs. 156.0 ± 72.5 respectively). Also, the majority of fibroids in both groups remained unchanged throughout the study period (17/31, 54.8% and 19/31, 61.3%). When analyzing the bleeding patterns at 3 months, results showed that women in groups A and C had a lower incidence of amenorrhea (56.5% and 51.4%) when compared to the placebo arm (87.1%) and complained more frequently of AUB, which were also more severe than those in group B. However, at the sixth, ninth and 12th treatment cycles, the bleeding pattern was not significantly different between the three groups.

Yang et al. [38] conducted a prospective observational trial comparing the effects over 3 years of CEE 0.625 mg/day plus MPA 5 mg/day as opposed to no treatment on 72 postmenopausal women with uterine leiomyomas. Their findings demonstrated a slight trend towards myomas growth in postmenopausal women subjected to HRT in the first two years, without reaching a statistical significance compared to untreated women. During the third year of treatment, fibroids showed a mild volume reduction, again with no statistical significance. A relevant increase in fibroid size (more than 25% compared to baseline values) was observed only in 8.1% of HRT-treated women for a period of 3 years.

A large population composed of 159 women at least 2 months from their last spontaneous menstruation was studied by Ylostalo and colleagues [36]. They were divided into three study groups as follows: group 1 consisted of women who had been menopausal for less than 3 years and received 1 mg transdermal estradiol plus MPA 10 mg/day for 12 days every month; group 2 was composed of women at least 3 years postmenopausal, who received the same treatment as above; group 3 was also composed of women 3 years postmenopausal, subjected to the same estradiol dosage, but receiving MPA 10 mg/day for 12 days every 3 months. Eighty patients out of 159 were diagnosed with uterine fibroids >8 mm in diameter by transvaginal ultrasound. According to their data, myomas diameters increased overall by 26% during the first 6 months of HRT, compared to baseline values, but no further enlargement was detected over the last 6 months.

Schwartz and colleagues [37] published a retrospective case series including 14 women with a total of 23 uterine fibroids treated with different HRT regimens, either 0.625 mg CEE daily, 1 mg micronized estradiol daily or 50 mcg transdermal estradiol daily. As for progestins, patients received either MPA 10 mg daily for 12 days every month or MPA 2.5 mg daily continuously. The authors also selected seven women with a total of eight uterine fibroids who rejected HRT to serve as the control group. The mean follow-up period was 19.7 ± 6.3 months in the HRT group and 9.7 ± 1.7 months in the control one. Data from this study did not demonstrate any significant changes in mean myoma volume from baseline within each group, regardless of the HRT regimen. On the other hand, 17 out of the 23 myomas (73.9%) in the hormonally-treated patients increased during the study period, as opposed to the growth of only two fibroids in the control group (25%). Interestingly, the authors found within the same patient one myoma that grew and one that shrank over the study period, suggesting that relevant biological differences in myomas even in the same uterus.

Lastly, Chang et al. [18] recently studied the effects of different kinds of HRT on 32 patients with asymptomatic uterine fibroids, as opposed to 6 women who did not receive hormonal treatment. Women in the study group received either 0.625 mg CEE daily, or eastradiol-17-valerate, or transdermal estrogen (both via transdermal patch–50 mcg 17-β-estradiol daily–or gel–1.5 mg 17-β-estradiol daily). As for progestins, MPA 5 mg or micronized progesterone 200 mg were employed, either with a continuous combined method or a sequential cyclic method for 12 days a month. Myomas volume was assessed every 6 months for a period of one year. After 6 months, myomas in both groups increased in size, but the change was not statistically significant when compared to the baseline values. Again, no differences were detected based on the prescription method of estrogen or the progestogen protocol. A significant enlargement (more than 30% of change in volume) was diagnosed in 28.1% of patients. The only significant difference found by this study was noted between the oral estrogen method and the transdermal estrogen one: in fact, myomas in the oral estrogen group showed no change in 45.5% of patients, as opposed to only 10% of patients in the transdermal group, where up to 50% of cases showed an enlargement of myomas over the first 6 months.

### 3.3. The Role of Progestins

In a prospective trial by Sener and colleagues [14], 40 menopausal women with at least one fibroid were randomized to receive two different HRT protocols: 22 patients in group I received transdermal estradiol 50 mcg/daily plus MPA 5 mg daily continuously, whereas 18 patients in group II were administered oral CEE 0.625 mg daily plus MPA 2.5 mg daily continuously. The size of fibroids was assessed before the treatment and after 12 months of HRT. Fibroids of women in group I showed a significant enlargement compared to baseline after 1 year (14.3 ± 5.9 mm vs. 19.7 ± 12.4 mm), while their diameter remained constant in group II (15.8 ± 6.3 mm vs. 15.7 ± 6.7 mm). Despite this, patients in group I still remained asymptomatic, and there was no difference in terms of spotting frequency and severity between the two groups. Moreover, the mean serum estradiol levels were significantly higher in both groups, with no relevant differences between the two treatment methods.

Polatti and colleagues [35] studied the effects of higher doses of progestins in a large cohort of 224 postmenopausal women. Among them, 150 did not have leiomyomas (groups A and B), whereas 74 were diagnosed with at least one fibroid measuring 2 to 4 cm on ultrasound (groups C and D). The four groups were further randomized into two treatment arms: groups A and C were administered combination of single-phase cyclic oral estradiol valerate 2 mg daily plus cyproterone acetate 1 mg daily for 21 days, whereas women in groups B and D received a cyclic schedule of transdermal estradiol 50 mcg/day for 21 days and MPA 10 mg/day from days 10 to 21 of cycle. The study period was 24 months, with transvaginal ultrasound evaluation at baseline and every 12 months. The combination of estradiol valerate and cyproterone acetate did not cause new myoma formation (group A), whereas 4 patients subjected to transdermal estradiol and MPA were newly diagnosed with leiomyomas after 12 months, with a mean size of 25.4 ± 1.2 cm^3^. Furthermore, while a non-significant increase in myoma volume at 12 months was noted in group C (18.6 ± 1.4 vs. 19.2 ± 1.1 cm^3^), myomas in women subjected to transdermal estradiol and MPA grew significantly after the first year of treatment (19.3 ± 1.3 vs. 23.8 ± 0.9 cm^3^). Uterine fibroids showed a mean volume increase of 4.8% in group C and of 25.5% in group D.

In the wake of this study and suspecting a central role of the progestin dose in the growth of fibroids in postmenopausal women, Palomba and colleagues [40] conducted a randomized controlled trial, enrolling 27 women with one or two leiomyomas with a mean diameter > 20 mm. Fourteen women in group A received oral micronized estradiol 2 mg/day plus MPA 2.5 mg/day, whereas women in group B received the same estrogen dose, but a doubled dose of MPA. Myoma dimensions (mm), incidence of amenorrhea and bleeding patterns (number and severity of AUB) were recorded every three months for one year. Myomas of patients who received the higher dosage of MPA showed a significant increase after 12 months, growing from 169.7 ± 85.1 to 201.3 ± 93.8 mm, whereas those in group A did not show statistically significant changes. Additionally, the incidence of amenorrhea and the number and severity of AUB resulted significantly different in both groups during the first 3 months, compared to those recorded at 6, 9 and 12-month evaluations, and bleeding patterns were overall similar between the two groups.

### 3.4. SERM

SERMs are a group of compounds that are able to elicit tissue- and target-specific responses, acting as estrogen receptors agonists or antagonists [42]. Having demonstrated a good tolerability profile and being effective in treating many climacteric symptoms, they have lower discontinuation rates than HRT [43,44]. However, their impact on uterine fibroids is still largely unknown, especially in menopausal women.

Raloxifene is a second-generation SERM which exerts an estrogen antagonistic activity on the breast and the endometrium, but acts as an estrogen agonist in the bone. Its influence on fibromatous tissue was investigated in two studies by Palomba and colleagues [31,32].

In their first experience, the authors enrolled 62 postmenopausal women with 1-2 leiomyomas measuring > 20 mm; 31 of them were subjected to raloxifene 60 mg/day, whereas 31 received placebo. After one year, leiomyomas volume in patients treated with raloxifene were significantly reduced in volume compared to the control group. Particularly, after 12 treatment courses a significant reduction in myomas size was noted in 83.9% of women. On the other hand, amenorrhea incidence and number and severity of AUB was comparable between the two groups.

In another prospective randomized double-blind trial [32], the authors enrolling 40 postmenopausal women with one to two intramural leiomyomas. Patients were allocated to receive either raloxifene hydrochloride 180 mg/day or placebo. The study followed patients for 3 cycles of 28 days. At the end of the study, fibroids in patients who received raloxifene showed a significant size reduction (141.7 ± 37.8 vs. 116.3 ± 27.4 cm^3^) both compared to baseline values and fibroids size in the control group, who were practically unchanged (150.3 ± 58.7 vs. 150.4 ± 58.0 cm^3^).

## 4. Discussion

Uterine leiomyomas are the most common benign neoplasm of the female reproductive organs. Although their exact etiology remains uncertain, the expression of estrogen and progesterone receptors in their tissue has been demonstrated by several studies [18,21,22,23] and the influence of sexual steroids on their formation and growth has long been hypothesized. During menopause, leiomyomas appear to shrink in volume, easing their burden on symptomatic women, who often require no treatment. In light of this, the use of HRT in women affected by fibroids has been debated, and many practitioners are still unwilling to prescribe HRT in this subset of patients.

This state-of-the-art review aimed to clarify the influence of different kinds of HRT on fibroids growth and fibroids-related symptoms after menopause, verifying whether a specific treatment method could be more appropriate in this instance.

Of the seventeen eligible studies found by our research, the majority had a prospective design, with nine being randomized controlled trials, whereas only two were retrospective studies. On the other hands, these studies were mostly conducted in the late 90s, had a small population and lacked a rigorous statistical analysis, having no power analysis, no clear randomization criteria and sometimes investigating different types of HRT at the same time. Moreover, many of these papers had a short-term follow-up, usually just 12 months. Thus, evidence from these studies should be taken with caution, making it difficult to draw general conclusion applicable to a large population.

Five studies investigated the effects of tibolone, a synthetic steroid which shows androgenic, estrogenic and progestogenic actions. In particular, three studies compared its influence on uterine fibroids with different regimens of HRT [28,29,30]. From the analysis of these data, tibolone appears to have no significant effect on myomas growth compared to placebo or estrogen-progestin therapy. Moreover, it is generally associated with fewer episodes of irregular spotting. However, only Fedele et al. [29] found a significant difference in terms of fibroids growth between tibolone-treated women and patients treated with estrogen-progestin therapy.

As for HRT, results are often conflicting. Some combinations of estrogen and progestin have exhibited a significant influence on fibroids enlargement, as well as in frequency of newly detected myomas in menopause [14,35,39,40]. By contrast, several studies failed to demonstrate a significant increase in fibroids size, although a trend towards enlargement was noted [18,33,34,37,38].

Interestingly, some of the studies that found a significant increase in fibroids dimensions used different progestin compounds [14,35,40], often using MPA at different doses [14,40], confirming the pivotal role of progesterone on leiomyomas growth. In particular, Sener et al. [14] administered different dosages of MPA but also different estrogens (one group receiving oral estrogen, one transdermal estrogen), whereas the study groups of Palomba and colleagues [40] differed only in terms of MPA dose. However, both authors concluded that fibroids size was more likely due to the different progestogen doses rather than the different estrogens, and this is further supported by the fact that Sener et al. found similar serum estradiol levels in the two study groups [14]. Furthermore, Fedele and colleagues [29] reported not only a significant enlargement of fibroids in the group of patients subjected to a high dose of MPA (10 mg), but also of the mean number of fibroids after 12 months.

Some efforts have been made to try to discover a possible marker for fibroids growth during HRT. According to Colacurci et al. [33], uterine artery pulsatility index at baseline could be a useful tool in predicting the risk of leiomyomas growth. In fact, the low resistance index found in uterine arteries of women with fibroids was correlated to a higher risk of fibroids growth during HRT. On the other hand, Gregoriou et al. [19] did not show any relevant correlation between PI values at baseline and the risk of myomas growth, arguing that PI could not be effectively relied upon.

As for SERMs, raloxifene has demonstrated an inhibitory effect on fibromatous tissue [31,32], both in animals and humans. However, only two studies investigated its effects on leiomyomas, and both for a short period of time.

## 5. Conclusions

To date, a wide variety of therapeutic options are available to treat menopause-related symptoms. The choice of the most appropriate therapy is crucial for women with uterine fibroids, especially those who were symptomatic and who already have large fibroids. Given the unevenness of available data, it could be argued that uterine myomas might be influenced by HRT, without representing an absolute contraindication to treatment. Indeed, several authors encourage the use of HRT in postmenopausal women with fibroids. However, these patients should be regularly subjected to a thorough follow-up, including transvaginal ultrasound for the monitoring of myomas size, and HRT should be discontinued if an increase in size of uterine fibroids is documented. Moreover, according to some authors it would be better to administer the minimal effective dose of progestin during HRT, to minimize the risk of fibroids growth.

## Figures and Tables

**Table 1:** Selected articles presented in detail.

Author	Study Design (Duration of HRT) and Objective	Patients	Type of HRT	Baseline	Follow-Up	*p* Value	Other Findings
**Chang et al. (2013)** [18]	Retrospective observational study (12 months) Myoma volume assessed every 6 months	38 women with uterine fibroids HRT (*n* = 32) No HRT (*n* = 6)	Oral estrogen (*n* = 22) 0.625 mg CEE daily Estradiol-17-valerate Transdermal estrogen (*n* = 10) 50 mcg 17-β-estradiol patch 1.5 mg 17-β-estradiol gel MPA 5 mg Micronized progesterone 200 mg Continuous combined pattern (*n* = 16) Sequential cyclic pattern (*n* = 16)	**Myoma volume (cm^3^)** Control: 17.6 ± 26.8 HRT: 19.5 ± 24.5	**Myoma volume (cm^3^)** **6 months**		Increase in size HRT: 9/32 (28.1%) No change in size HRT: 13/32 (40.6%) Decrease in size HRT: 10/32 (31.3%)
					Control: 16.5 ± 23.2	NS	
					HRT: 24.7 ± 35.1	NS	
					**12 months**		
					Control: 21.1 ± 34.6	NS	
					HRT: 28.5 ± 56.4	NS	
**Colacurci et al. (2000)** [33]	Prospective observational study (12 months) Myoma size and uterine arteries pulsatility index (PI)	60 menopausal on HRT Group A (*n* = 20). No uterine fibroids Group B (*n* = 15) Single asymptomatic fibroid < 3 cm, IM or SS - B1: quiescent myoma - B2: myoma growing >20% Group C (*n* = 18) Single asymptomatic fibroid >3 cm, IM or SS - C1: quiescent myoma - C2: myoma growing >20%	Continuous transdermal 17-beta-estradiol 0.05 mg/day plus nomegestrol acetate 5 mg/day sequentially added (days 17 to 28)	**Myoma volume (cm^3^)** B + C: 24.1 ± 20.0 **Uterine artery PI** A: 2.4 ± 0.5* B: 1.7 ± 0.2* C: 1.6 ± 0.3*	**Myoma volume (cm^3^)** **3 months**		Increased in volume B: 2/33 (6.1%) C: 6/33 (18.1%) Total: 8/33 (24.2%)
					26.1 ± 22.3	NS	
					**6 months**		
					26.6 ± 25.6	NS	
					**9 months**		
					27.8 ± 28.0	NS	
					**12 months**		
					28.8 ± 30.0	NS	
					**Uterine artery PI** **3 months**		
					A: 2.2 ± 0.3	NS	
					B: 1.9 ± 0.2 #	<0.01	
					C: 1.9 ± 0.2 #	<0.01	
					**6 months**		
					A: 2.3 ± 0.3	NS	
					B: 1.9 ± 0.2		
					C: 1.9 ± 0.2		
					**9 months**	NS	
					A: 2.2 ± 0.3		
					B: 1.9 ± 0.2		
					C: 1.9 ± 0.2		
					**12 months**	NS	
					A: 2.2 ± 0.3		
					B: 1.9 ± 0.2		
					C: 1.9 ± 0.2		
**De Aloysio et al. (1998)** [28]	Prospective randomized controlled trial (12 months) Myoma size and bleeding patterns	50 women, 47 completed the study 1 to 4 asymptomatic submucous or intramural leiomyomas with the longest diameter ranging from 3 to 8 cm	Group A (*n* = 24): Tibolone 2.5 mg/day Group B (*n* = 23): CEE 0.625 mg/day plus MPA 5 mg/day.	NA	NA—no difference in terms of size		Irregular bleeding incidence A: 22.6% of cycles B: 29.7% of cycles Irregular spotting incidence A: 2.4% B: 4.7% Bleeding and spotting lengths did not differ between the two groups
**Fedele et al. (2000)** [29]	Prospective randomized controlled trial (12 months) Uterine and myoma size, myoma number. Symptoms and menstrual patterns.	38 women with 1 or more uterine myomas, at least one with a diameter ≥3 cm.	Group A (*n* = 20): Transdermal estradiol-releasing system, 50 mcg/day + oral MPA 10 mg/day for 12 days/month Group B (*n* = 18): Tibolone 2.5 mg/day.	**No. of myomas** A: 1.75 ± 0.79 B: 1.62 ± 0.82 **Volume of largest (cm^3^)** A: 26.8 ± 12.5 B: 28.1 ± 14.1	**No. of myomas** **6 months**		
					A: 1.95 ± 0.94	NS	
					B: 1.70 ± 0.91	NS	
					**12 months**		
					A: 2.15 ± 0.93 #	<0.01	
					B: 1.67 ± 0.85	NS	
					**Volume of largest (cm^3^)**		
					**6 months**		
					A: 32.5 ± 14.2 #	<0.01	
					B: 30.2 ± 12.8	NS	
					**12 months**		
					A: 35.8 ± 0.17 #	<0.01	
					B: 30.6 ± 16.2	NS	
**Frigo et al. (1995)** [39]	Prospective observational trial (12 months) Myoma size and number	50 perimenopausal women	4 mg estradiol-valerate + 200 mg prasteronenantate intramuscular injections every 6-10 weeks	**Mean diameter (mm)**	**Mean diameter (mm)**		
				29.4 mm	35.0 mm	<0.01	
				**Mean number**	**Mean number**		
				2.2.	3.5	NS	
**Gregoriou et al. (1997)** [19]	Prospective randomized controlled trial (12 months) Myoma size	40 women with at least 1 myoma measuring >20 mm	Group A (*n* = 20): Tibolone 2.5 mg/day Group B (*n* = 20): no treatment	**Myoma volume (cm^3^)**	**Myoma volume (cm^3^)** **12 months**		Increase in size A: 3/20 (15%) B: 2/20 (10%) Constant size A: 14/20 (70%) B: 15/20 (75%) Decrease in size A: 3/20 (15%) B: 3/20 (15%)
				A: 102.6	A: 98.2	NS	
				B: 118.4	B: 117.5	NS	
**Gregoriou et al. (2001)** [27]	Prospective observational study (36 months) Myoma size and uterine arteries PI	66 women Group A (*n* = 20) No myomas Group B (*n* = 23) Single asymptomatic IM-SS myoma ≤2 cm Group C (*n* = 23) Single asymptomatic IM-SS myoma 2–5 cm	Tibolone 2.5 mg/day	**Myoma volume (cm^3^)** B: 15.8 ± 1.4 C: 28.2 ± 1.6 **Uterine artery PI** A: 2.44 ± 0.35 * B: 1.62 ± 0.25 * C: 1.72 ± 0.23 *	**Myoma volume**		Increase in size B: 2/23 (8.7%) C: 3/23 (13.1%) Constant size B: 21/23 (91.3%) C: 20/23 (86.9%)
					NA		
					**Uterine artery PI** **6 months**		
					A: 2.25 ± 0.31 #	<0.01	
					B: 1.83 ± 0.21 #	<0.01	
					C: 1.91 ± 0.22 #	<0.01	
					**36 months**		
					A: 2.22 ± 0.29	NS	
					B: 1.86 ± 0.20	NS	
					C: 1.94 ± 0.20	NS	
**Palomba et al. (2001)** [34]	Prospective nonrandomized observational trial (12 months) Myomas size Uterine bleeding pattern	92 women Group A (*n* = 31) With 1 or 2 IM-SS myomas > 20 mm Group B (*n* = 31) With 1 or 2 IM-SS myomas > 20 mm Group C (*n* = 30) No leiomyomas	Group A Transdermal estradiol patches (50 mcg/day) plus oral MPA (2.5 mg/day), continuously added Group B Placebo (calcium carbonate) Group C Transdermal estradiol patches (50 mcg/day) plus oral MPA (2.5 mg/day), continuously added	**Myoma volume (cm^3^)** A: 141.7 ± 37.8 B: 150.3 ± 58.7	**Myoma volume (cm^3^)** **12 months**		Increase in size A: 8/31 (25.8%) B: 6/31 (19.4%) Unchanged A: 17/31 (54.8%) B: 19/31 (61.3%) Decreased in size A: 6/31 (19.4%) B: 6/31 (19.4%)
					A: 147.5 ± 53.3	NS	
					B: 156.0 ± 72.5	NS	
					**Amenorrhea (incidence)** **3 months**		
					A: 56.5% §	<0.05	
					B: 87.1%	NS	
					C: 51.4% §	<0.05	
					**12 months**		
					A: 76.8%	NS	
					B: 86.0%	NS	
					C: 76.4%	NS	
					**N of AUB** **3 months**		
					A: 0.54 ± 0.75 §	<0.05	
					B: 0.22 ± 0.21	NS	
					C: 0.61 ± 0.74 §	<0.05	
					**12 months**		
					A: 0.23 ± 0.42	NS	
					B: 0.19 ± 0.19	NS	
					C: 0.24 ± 0.43	NS	
					**Severity of AUB** **3 months**		
					A: 1.51 ± 0.56 §	<0.05	
					B: 0.92 ± 0.23	NS	
					C: 1.64 ± 0.68 §	<0.05	
					**12 months**		
					A: 1.06 ± 0.25	NS	
					B: 0.86 ± 0.22	NS	
					C: 1.05 ± 0.24	NS	
**Palomba et al. (2002)** [40]	Prospective randomized controlled trial (12 months) Myomas size Uterine bleeding pattern	27 women with 1–2 uterine leiomyomas > 20 mm	Group A (*n* = 14) Oral micronized estradiol 2 mg/day plus MPA 2.5 mg/day Group B (*n* = 13) Oral micronized estradiol 2 mg/day plus MPA 5 mg/day	**Myoma dimensions (mm)** A: 186.6 ± 77.6 B: 169.7 ± 85.1	**Myoma dimensions (mm)**		Increased in size A: 3/14 (21.4%) B: 7/13 (53.8%) Unchanged A: 8/14 (57.1%) B: 4/13 (30.8%) Decreased in size A: 3/13 (23.1%) B: 2/14 (14.3%)
					**12 months**		
					A: 198.6 ± 97.6	NS	
					B: 201.3 ± 93.8 *	<0.05	
					**Amenorrhea**		
					**3 months**		
					A: 51.3% §	<0.05	
					B: 52.4% §	<0.05	
					**12 months**		
					A: 76.9%	NS	
					B: 80.9%	NS	
					**N of AUB**		
					**3 months**		
					A: 0.59 ± 0.81 §	<0.05	
					B: 0.63 ± 0.86 §	<0.05	
					**12 months**		
					A: 0.21 ± 0.41	NS	
					B: 0.24 ± 0.43	NS	
					**Severity of AUB**		
					**3 months**		
					A: 1.53 ± 0.71 §	<0.05	
					B: 1.58 ± 0.66 §	<0.05	
					**12 months**		
					A: 1.12 ± 0.32	NS	
					B: 1.13 ± 0.35	NS	
**Palomba et al. (2001)** [31]	Prospective randomized double-blind trial (12 months: 12 cycles of 28 days) Myoma size Amenorrhea AUB number and severity	62 postmenopausal women with 1-2 leiomyomas measuring > 20 mm	Group A (*n* = 31) Raloxifene 60 mg/day Group B (*n* = 31) Placebo	**Myoma volume (cm^3^)** A: 127.1 ± 38.2 B: 138.6 ± 55.7	**Myoma volume (cm^3^)** NA, significantly reduced Amenorrhea incidence and AUB number and severity: comparable within and between groups	<0.05	Decreased in size 3 cycles 1/31 (3.2%) 6 cycles 13/31 (41.9%) 9 cycles 24/31 (77.4%) 12 cycles 26/31 (83.9%)
**Palomba et al. (2005)** [32]	Prospective randomized double-blind trial (84 days: 3 cycles of 28 days) Myoma size	40 postmenopausal women with 1-2 intramural uterine leiomyomas, one measuring >2 cm	Treatment group (TG) (*n* = 20) Raloxifene hydrochloride 180 mg/day Control group (CG) (*n* = 20) Placebo	**Myoma volume (cm^3^)** TG: 141.7 ± 37.8 CG: 150.3 ± 58.7	**Myoma volume (cm^3^)** TG: 116.3 ± 27.4 CG: 150.4 ± 58.0 P = 0.022 (between TG and CG)	<0.05	Mean change in leiomyoma size: TG: -17.4 ± 6.1 CG: 1.9 ± 1.1
**Polatti et al. (2000)** [35]	Prospective randomized controlled trial (24 months) Myoma size and new formation of fibroids	224 women No myomas Group A (*n* = 76) Group B (*n* = 74) Myomas Group C (*n* = 38) Group D (*n* = 36)	Single-phase cyclic estradiol valerate 2 mg/day and cyproterone acetate 1 mg/day for 21 days + 7-day break. Groups A and C Combined sequential cyclic transdermal estradiol 50 mcg/day and MPA 10 mg/day days 10-21, 7-day break. Groups B and D.	**Myoma volume (cm^3^)** C: 18.6 ± 1.4 D: 19.3 ± 1.3	**Myoma volume (cm^3^)**		Group B In 4 women (5.4%) new onset of fibromas, mean size 25.4 ± 1.2 Group D Mean increase in myoma volumes of 25.3% after 24 months Group C Mean increase in myoma volumes of 4.8% after 24 mo.
					**12 months**		
					C: 19.2 ± 1.1 #	<0.01	
					D: 23.8 ± 0.9 * #	<0.01	
					**24 months**		
					C: 19.5 ± 1.1 #	<0.01	
					D: 24.2 ±0.8 * #	<0.01	
**Schwartz et al. (1996)** [37]	Retrospective case series report (HRT 19.7 ± 6.3 months, Controls 9.7 ± 1.7 months) Myoma size	Case group 14 cases with uterine leiomyomas treated with HRT (23 myomas) Control group 7 cases with uterine leiomyomas untreated (8 myomas)	CEE 0.625 mg/day (*m* = 7) Micronized estradiol 1 mg/day (*n* = 5) Transdermal 50 mcg estradiol patch (*n* = 2). MPA (*n* = 11) Micronized progesterone (*n* = 3) Cyclical regimen (MPA 10 mg for 12 days/month) (*n* = 10) Continuous combined (MPA 2.5 mg/day) (*n* = 4)	**Myoma volume (cm^3^)**	**Myoma volume (cm^3^)**		Mean change in myoma volume Case: +13.9 ± 8.2 Control: -2.0 ± 1.9 Increase in size Case: 17/23 (73.9%) Control: 2/8 (25%) Decrease in size Case: 6/23 (26.1%) Control: 5/8 (62.5%) Unchanged Control: 1/8 (12.5%)
				Case: 15.7 ± 5.5	Case: 29.6 ± 12.2	NS	
				Control: 4.5 ± 2.5	Control: 2.5 ± 1.1	NS	
**Sener et al. (1996)** [14]	Prospective randomized controlled trial (12 months) Myoma size	40 menopausal women with at least one myoma <20 mm	Group I (*n* = 22) Transdermal 50 mcg/day estradiol + 5 mg MPA continuously Group II (*n* = 18) Oral CEE 0.625 mg + 2.5 mg MPA continuously	**Myoma diameter (mm)**	**Myoma diameter (mm)**		No difference in E2 levels between the two groups
				I: 14.3 ± 5.9	I: 19.7± 12.4 #	<0.05	
				II: 15.8 ± 6.3	II: 15.7 ± 6.7	NS	
**Simsek et al. (2002)** [30]	Prospective randomized controlled trial (6 months) Myoma size	46 women with leiomyomas	Group A (*n* = 24, 32 myomas) Tibolone 2.5 mg/day Group B (*n* = 22, 28 myomas) Transdermal estradiol 0.05 mg/day for 4 weeks + norethisterone acetate 0.25 mg/day for 2 weeks	**Myoma volume (cm^3^)**	**Myoma volume (cm^3^)**		Increased in size A: 4/32 (12.5%) NS B: 6/28 (21.4%)
				A: 18.6 ± 4.1	A: 20.1 ± 4.0	NS	
				B: 23.1 ± 3.6	B: 27.2± 3.9	NS	
**Yang et al. (2002)** [38]	Prospective observational trial (36 months) Myoma size	72 women with leiomyomas > 20 mm	HRT group (*n* = 37) CEE 0.625 mg/day + MPA 5 mg/day Control group (*n* = 35) No therapy	**Myoma volume (cm^3^)** HRT: 21.7 ± 11.7 Controls: 19.4 ± 8.7	**Myoma volume (cm^3^)**		Increased in size 1st year HRT: 4/37 (10.8%) NS Controls: 2/35 (5.7%) 2nd year HRT: 5/37 (13.5%) Controls: 2/35 (5.7%) 3rd year HRT: 3/37 (8.1%) Controls: 1/35 (2.9%)
					**1st year**		
					HRT: 23.2 ± 12.5		
					Controls: 20.4 ± 9.4	NS	
					**2nd year**		
					HRT: 23.7 ± 12.8	NS	
					Controls: 20.7 ± 9.4		
					**3rd year**		
					HRT: 23.5 ± 12.8	NS	
					Controls: 20.1 ± 9.3		
**Ylostalo P et al. (1996)** [36]	Prospective observational trial (12 months) Myoma size	159 patients–80 studied by TVS–19 had myomas >8 mm	Group 1 (2 months-3 years postmenopausal) 1 mg transdermal estradiol + 10 mg MPA/day for 12 days every month Group 2 (> 3 years postmenopausal) 1 mg transdermal estradiol + 10 mg MPA/day for 12 days every month Group 3 (> 3 years postmenopausal) 1 mg transdermal estradiol + 10 mg MPA/day for 12 days every 3 months	**Myoma diameter (mm)** 1: 14.5 ± 5.2 2: 17.0 ± 7.2 3: 17.0 ± 6.6	**Mean diameters (mm)** Mean increase: 26% over the first 6 months, no further increase during the last 6 months	NA	

* Significant difference between study groups. # Significant difference from baseline within the same study group. § Significant difference within the same study group compared to 6th, 9th and 12th cycle of treatment.

## References

[1] Moravek M.B., Bulun S.E. (2015). Endocrinology of Uterine Fibroids: Steroid Hormones, Stem Cells, and Genetic Contribution. Curr. Opin. Obstet. Gynecol..

[2] Seracchioli R., Colombo F., Bagnoli A., Govoni F., Missiroli S., Venturoli S. (2003). Laparoscopic myomectomy for fibroids penetrating the uterine cavity: Is it a safe procedure?. BJOG Int. J. Obstet. Gynaecol..

[3] Seracchioli R., Manuzzi L., Vianello F., Gualerzi B., Savelli L., Paradisi R., Venturoli S. (2006). Obstetric and delivery outcome of pregnancies achieved after laparoscopic myomectomy. Fertil. Steril..

[4] Seracchioli R. (2000). Fertility and obstetric outcome after laparoscopic myomectomy of large myomata: A randomized comparison with abdominal myomectomy. Hum. Reprod..

[5] Townsend D.E., Sparkes R.S., Baluda M.C., McClelland G. (1970). Unicellular histogenesis of uterine leiomyomas as determined by electrophoresis by glucose-6-phosphate dehydrogenase. Am. J. Obstet. Gynecol..

[6] Wise L.A., Palmer J.R., Harlow B.L., Spiegelman D., Stewart E.A., Adams-Campbell L.L., Rosenberg L. (2004). Reproductive Factors, Hormonal Contraception, and Risk of Uterine Leiomyomata in African-American Women: A Prospective Study. Am. J. Epidemiol..

[7] Marshall L.M., Spiegelman D., Goldman M.B., E Manson J., Colditz G.A., Barbieri R.L., Stampfer M.J., Hunter D.J. (1998). A prospective study of reproductive factors and oral contraceptive use in relation to the risk of uterine leiomyomata. Fertil. Steril..

[8] Laughlin S.K., Schroeder J.C., Baird D.D. (2010). New Directions in the Epidemiology of Uterine Fibroids. Semin. Reprod. Med..

[9] Frascà C., Tuzzato G., Arena A., Degli Esposti E., Zanello M., Raimondo D., Seracchioli R. (2018). The Role of Pelvic Ultrasound in Preoperative Evaluation for Laparoscopic Myomectomy. J. Minim. Invasive Gynecol..

[10] Frascà C., Degli Esposti E., Arena A., Tuzzato G., Moro E., Martelli V., Seracchioli R. (2018). Can In-Bag Manual Morcellation Represent an Alternative to Uncontained Power Morcellation in Laparoscopic Myomectomy? A Randomized Controlled Trial. Gynecol. Obstet. Investig..

[11] Casadio P., Guasina F., Morra C., Talamo M.T., Leggieri C., Frisoni J., Seracchioli R. (2016). Hysteroscopic myomectomy: Techniques and preoperative assessment. Minerva Ginecol..

[12] Seracchioli R., Venturoli S., Colombo F., Bagnoli A., Vianello F., Govoni F., Guerrini M., Gualerzi B. (2003). GnRH Agonist Treatment before Total Laparoscopic Hysterectomy for Large Uteri. J. Am. Assoc. Gynecol. Laparosc..

[13] Segars J.H., Parrott E.C., Nagel J.D., Guo X.C., Gao X., Birnbaum L.S., Pinn V.W., Dixon D. (2014). Proceedings from the Third National Institutes of Health International Congress on Advances in Uterine Leiomyoma Research: Comprehensive review, conference summary and future recommendations. Hum. Reprod. Updat..

[14] Sener A.B., Seçkin N.C., Ozmen S., Gökmen O., Doğu N., Ekici E. (1996). The effects of hormone replacement therapy on uterine fibroids in postmenopausal women. Fertil. Steril..

[15] Wu J., Cheng Y. (1995). Research on the relationship between estrogen receptor, progesterone receptor, cell proliferation associated antigen in uterine leiomyoma and nuclear body density of myoma, serum reproductive hormone concentrations. Zhonghua fu Chan ke za Zhi.

[16] Rein M.S. (2000). Advances in Uterine Leiomyoma Research: The Progesterone Hypothesis. Environ. Health Perspect..

[17] Shozu M., Murakami K., Inoue M. (2004). Aromatase and Leiomyoma of the Uterus. Semin. Reprod. Med..

[18] Chang I.J., Hong G.Y., Oh Y.L., Kim B.R., Park S.N., Lee H.-H., Na Y.-J., Namkung J. (2013). Effects of Menopausal Hormone Therapy on Uterine Myoma in Menopausal Women. J. Menopausal Med..

[19] Gregoriou O., Vitoratos N., Papadias C., Konidaris S., Costomenos D., Chryssikopoulos A. (1997). Effect of tibolone on postmenopausal women with myomas. Maturitas.

[20] Cermik D., Arici A., Taylor H.S. (2002). Coordinated regulation of HOX gene expression in myometrium and uterine leiomyoma. Fertil. Steril..

[21] Srinivasan V., Martens M.G. (2018). Hormone therapy in menopausal women with fibroids: Is it safe?. Menopause.

[22] Brandon D.D., Bethea C.L., Strawn E.Y., Novy M.J., Burry K.A., Harrington M.S., Erickson T.E., Warner C., Keenan E.J., Clinton G.M. (1993). Progesterone receptor messenger ribonucleic acid and protein are overexpressed in human uterine leiomyomas. Am. J. Obstet. Gynecol..

[23] Nisolle M., Gillerot S., Casanas-Roux F., Squifflet J., Berliere M., Donnez J. (1999). Immunohistochemical study of the proliferation index, oestrogen receptors and progesterone receptors A and B in leiomyomata and normal myometrium during the menstrual cycle and under gonadotrophin-releasing hormone agonist therapy. Hum. Reprod..

[24] Plewka D., Marczyński J., Morek M., Bogunia E., Plewka A. (2014). Receptors of Hypothalamic-Pituitary-Ovarian-Axis Hormone in Uterine Myomas. BioMed Res. Int..

[25] World Health Organization (1996). Research on the Menopause in the 1990s. Report of a WHO Scientific Group.

[26] Grisendi V., Spada E., Argento C., Plebani M., Milani S., Seracchioli R., Volpe A., La Marca A. (2014). Age-specific reference values for serum FSH and estradiol levels throughout the reproductive period. Gynecol. Endocrinol..

[27] Gregoriou O., Konidaris S., Botsis D., Papadias C., Makrakis E., Creatsas G. (2001). Long term effects of Tibolone on postmenopausal women with uterine myomas. Maturitas.

[28] De Aloysio D., Altieri P., Penacchioni P., Salgarello M., Ventura V. (1998). Bleeding patterns in recent postmenopausal outpatients with uterine myomas: Comparison between two regimens of HRT. Maturitas.

[29] Fedele L., Bianchi S., Raffaelli R., Zanconato G. (2000). A randomized study of the effects of tibolone and transdermal estrogen replacement therapy in postmenopausal women with uterine myomas. Eur. J. Obstet. Gynecol. Reprod. Biol..

[30] Simsek T., Karakus C., Trak B. (2002). Impact of different hormone replacement therapy regimens on the size of myoma uteri in postmenopausal period Tibolone versus transdermal hormonal replacement system. Maturitas.

[31] Palomba S., Sammartino A., Di Carlo C., Affinito P., Zullo F., Nappi C. (2001). Effects of raloxifene treatment on uterine leiomyomas in postmenopausal women. Fertil. Steril..

[32] Palomba S., Orio F., Russo T., Falbo A., Tolino A., Lombardi G., Cimini V., Zullo F. (2005). Antiproliferative and proapoptotic effects of raloxifene on uterine leiomyomas in postmenopausal women. Fertil. Steril..

[33] Colacurci N., De Franciscis P., Cobellis L., Nazzaro G., De Placido G. (2000). Effects of hormone replacement therapy on postmenopausal uterine myoma. Maturitas.

[34] Palomba S., Sena T., Noia R., Carlo C.D., Zullo F., Mastrantonio P. (2001). Transdermal Hormone Replacement Therapy in Postmenopausal Women with Uterine Leiomyomas. Obstet. Gynecol..

[35] Polatti F., Viazzo F., Colleoni R., Nappi R.E. (2000). Uterine myoma in postmenopause: A comparison between two therapeutic schedules of HRT. Maturitas.

[36] Ylöstalo P., Granberg S., Bäckström A.-C., Hirsjärvi-Lahti T. (1996). Uterine findings by transvaginal sonography during percutaneous estrogen treatment in postmenopausal women. Maturitas.

[37] Schwartz L.B., Simcha L., Meryl M., Nactigall L.E., Horan C., Goldstein S.R. (1996). Does the Use of Postmenopausal Hormone Replacement Therapy Influence the Size of Uterine Leiomyomata? A Preliminary Report. Menopause.

[38] Yang C., Lee J., Hsu S., Kuo C., Tsai E. (2002). Effect of hormone replacement therapy on uterine fibroids in postmenopausal women—A 3-year study. Maturitas.

[39] Frigo P., Eppel W., Asseryanis E., Sator M., Golaszewski T., Gruber D., Lang C., Huber J. (1995). The effects of hormone substitution in depot form on the uterus in a group of 50 perimenopausal women—A vaginosonographic study. Maturitas.

[40] Palomba S., Sena T., Morelli M., Noia R., Zullo F., Mastrantonio P. (2002). Effect of different doses of progestin on uterine leiomyomas in postmenopausal women. Eur. J. Obstet. Gynecol. Reprod. Biol..

[41] Huang K.-E., Baber R. (2010). Updated clinical recommendations for the use of tibolone in Asian women. Climacteric.

[42] An K.-C. (2016). Selective Estrogen Receptor Modulators. Asian Spine J..

[43] Del Pup L. (2016). Ospemifene: A safe treatment of vaginal atrophy. Eur. Rev. Med. Pharmacol. Sci..

[44] Johnson K., Hauck F. (2016). Conjugated Estrogens/Bazedoxifene (Duavee) for Menopausal Symptoms. Am. Fam. Physician.

